# Estimating the financial impact of livestock schistosomiasis on traditional subsistence and transhumance farmers keeping cattle, sheep and goats in northern Senegal

**DOI:** 10.1186/s13071-021-05147-w

**Published:** 2022-03-22

**Authors:** Praise Adeyemo, Elsa Léger, Elizabeth Hollenberg, Nicolas Diouf, Mariama Sène, Joanne P. Webster, Barbara Häsler

**Affiliations:** 1 Department of Pathobiology and Population Sciences, Royal Veterinary College, University of London, Hawkshead Lane, Hatfield, Hertfordshire, AL9 7TA UK; 2 London Centre for Neglected Tropical Disease Research, School of Public Health, Imperial College London, London, UK; 3 Institut Supérieur de Formation Agricole et Rurale, Université de Thiès, Bambey, Senegal; 4 Unité de Formation et de Recherche des Sciences Agronomiques, d’Aquaculture et de Technologies Alimentaires, Université Gaston Berger, Saint-Louis, Senegal; 5 Present Address: Dr Ameyo Stella Adadevoh (DRASA) Health Trust, Yaba, Lagos Nigeria

**Keywords:** Schistosomiasis, One Health, NTDs, Livestock, Subsistence farming, Praziquantel, Financial impact, Partial budget analysis, Senegal, Disease control

## Abstract

**Background:**

Schistosomiasis is a disease that poses major threats to human and animal health, as well as the economy, especially in sub-Saharan Africa (SSA). Whilst many studies have evaluated the economic impact of schistosomiasis in humans, to date only one has been performed in livestock in SSA and none in Senegal. This study aimed to estimate the financial impact of livestock schistosomiasis in selected regions of Senegal.

**Methods:**

Stochastic partial budget models were developed for traditional ruminant farmers in 12 villages in northern Senegal. The models were parameterised using data from a cross-sectional survey, focus group discussions, scientific literature and available statistics. Two scenarios were defined: scenario 1 modelled a situation in which farmers tested and treated their livestock for schistosomiasis, whilst scenario 2 modelled a situation in which there were no tests or treatment. The model was run with 10,000 iterations for 1 year; results were expressed in West African CFA francs (XOF; 1 XOF was equivalent to 0.0014 GBP at the time of analysis). Sensitivity analyses were conducted to assess the impact of uncertain variables on the disease costs.

**Results:**

Farmers surveyed were aware of schistosomiasis in their ruminant livestock and reported hollowing around the eyes, diarrhoea and weight loss as the most common clinical signs in all species. For scenario 1, the median disease costs per year and head of cattle, sheep and goats were estimated at 13,408 XOF, 27,227 XOF and 27,694 XOF, respectively. For scenario 2, the disease costs per year and head of cattle, sheep and goats were estimated at 49,296 XOF, 70,072 XOF and 70,281 XOF, respectively.

**Conclusions:**

Our findings suggest that the financial impact of livestock schistosomiasis on traditional subsistence and transhumance farmers is substantial. Consequently, treating livestock schistosomiasis has the potential to generate considerable benefits to farmers and their families. Given the dearth of data in this region, our study serves as a foundation for further in-depth studies to provide estimates of disease impact and as a baseline for future economic analyses. This will also enable One Health economic studies where the burden on both humans and animals is estimated and included in cross-sectoral cost–benefit and cost-effectiveness analyses of disease control strategies.

**Graphical Abstract:**

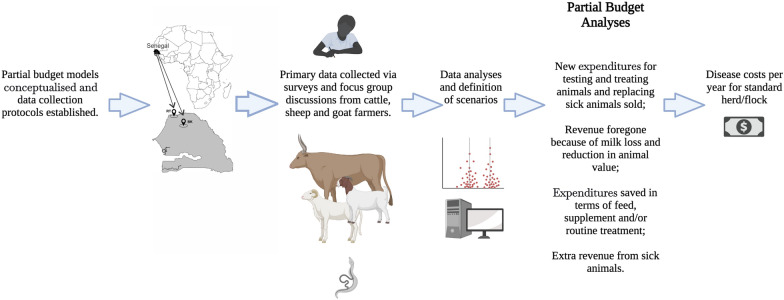

**Supplementary Information:**

The online version contains supplementary material available at 10.1186/s13071-021-05147-w.

## Background

Schistosomiasis is a major neglected tropical disease (NTD), second only to malaria as a parasitic disease of humans in terms of socio-economic impact [1]. The causative agents, *Schistosoma* spp., are dioecious trematodes which affect both humans and animals and are indirectly transmitted to their mammalian definitive hosts via freshwater molluscan intermediate hosts [2–4]. Over 240 million people are estimated to be infected with schistosomiasis caused by *Schistosoma haematobium* (and hybrids therein), *S. japonicum*, *S. mansoni*, *S. mekongi*, *S. guineensis* or *S. intercalatum* [5], with more than 90% of human cases occurring within sub-Saharan Africa (SSA) [3].

Whilst zoonotic transmission of schistosomiasis between humans and over 40 potential mammalian reservoir hosts is fully acknowledged within Asia [6–8], there is also an increasingly acknowledged zoonotic role within Africa [9, 10], as well as an awareness of the morbidity impact of animal schistosomiasis in general [11, 12]. Although the total number of livestock infected globally has not been accounted for [13], schistosomiasis in domestic animals often occurs within the same underprivileged communities most affected by human schistosomiasis [9, 11]. Furthermore, in addition to the previously assumed host-specific *Schistosoma* species, across many parts of SSA in particular, viable hybridised combinations including *S. haematobium*:*S. bovis*, *S. haematobium*:*S. curassoni*, *S. haematobium*:*S. mattheei* and *S. bovis*:*S. curassoni* have been reported in humans, while *S. bovis*, *S. curassoni* and *S. mattheei* together with *S. bovis*:*S. curassoni* and *S. bovis*:*S. mattheei* hybrids have been documented in domestic livestock [2, 14–17].

Since 2002, large-scale mass drug administration (MDA) with praziquantel (PZQ) as preventative chemotherapy in high-risk groups of children, predominantly school-age children, has been implemented across much of SSA [18]. Morbidity control has been generally successful across many countries [19] and has led to a revision of the World Health Organization’s (WHO’s) strategic plan for a vision of “a world free of schistosomiasis” in 2012 [20, 21], and more recently the new WHO NTD Road Map aimed at achieving elimination as a public health problem (EPHP), i.e. elimination of morbidity where the prevalence of heavy infection intensity in school-age children is less than 1% in all endemic countries by 2030, as well as a complete interruption of transmission (IoT, i.e. reduction in incidence of infection to zero) in selected African regions by the same point [22]. However, the sole focus of MDA in humans without complementary control of the disease in livestock, as well as misuse of the only available drug, PZQ, in animals to control livestock schistosomiasis, continues to frustrate efforts to achieve schistosomiasis control and elimination goals stipulated by the WHO within SSA [11].

Furthermore, schistosomiasis has been reported as one of the NTDs with the greatest unequal socioeconomic distribution [23], posing a threat to public health and having grave economic implications [24–26]. The drug PZQ is donated at a large scale by pharmaceutical companies, predominantly Merck KGA, and given for free to school-age children across many SSA countries [27] at an estimated value of $32.5 million annually [28]. Evaluations to date described the cost of the disease in humans in terms of disability-adjusted life years (DALYs), quality-adjusted life years (QALYs), the number of working days lost, and the financial burden of the disease [25]. Redekop et al. [29], for instance, conducted a review of studies on the economic impact of human schistosomiasis in terms of treatment costs and disease costs and estimated the global annual productivity loss associated with schistosomiasis at $5.5 billion from 2011 to 2020, and $11.9 billion from 2021 to 2030.

There is a dearth of studies, in contrast, on the economic implications of animal schistosomiasis [11]. A few studies have reported on the treatment costs for the disease to farmers and the biological effects and productivity impact of livestock schistosomiasis. They found that the different species of schistosomes cause organ pathologies in cattle [30], sheep [31] and goats [32], as well as productivity losses of meat, milk and reproduction [33]. To the authors’ knowledge, the only published study estimating the economic impact of schistosomiasis in animals in Africa is a benefit–cost analysis of investing in a potential vaccine for schistosomiasis in cattle in Sudan [33]. In this Sudanese study, the disease costs included production losses and the capital and operating costs of the vaccination programme. The benefit–cost ratios were estimated based on infection probability, vaccine uptake, mortality and vaccine production costs. The study showed that for every $1 spent on bovine schistosomiasis in provinces with a 50% infection probability, lower mortality, low vaccination and high vaccine production costs, the benefit–cost ratio was $0.7. However, in provinces with a high infection probability, high mortality rates, high percentage of vaccinated animals and low vaccine production costs, the benefits were higher, at $12.7, for every $1 invested [33]. These results showed that the development of cost-effective vaccines would yield high returns on investment.

The lack of economic assessments of livestock schistosomiasis makes decisions on investment in the treatment of livestock schistosomiasis difficult, particularly given the need to balance any potential benefits gained with increased risks in terms of the evolution of PZQ resistance [10], and where there might be other endemic disease priorities for the sector. Livestock schistosomiasis not only affects measures to control or eliminate human schistosomiasis but also causes disease costs for farmers, affects livelihoods and reduces the availability of livestock-derived foods for human consumption. Knowledge of the losses caused by the disease and expenditures needed for diagnosis and treatment enables the generation of a baseline of the current impact of the disease [34]. This baseline can then be used in cost–benefit or cost-effectiveness analyses to estimate the potential value of control strategies (e.g., mass or targeted drug treatment of animals) for individual farmers or the sub-sector.

The aim of this study was to estimate the financial impact of livestock schistosomiasis on traditional subsistence and transhumance farmers in selected villages around the Lac de Guiers and Barkedji town in Senegal. The objectives were to (1) establish herd/flock structures and production parameters for a regular cattle, sheep and goat herd or flock in northern Senegal, and (2) estimate losses and expenditures due to schistosomiasis in these production systems. The findings are discussed in terms of the potential economic impact livestock schistosomiasis can have on the livelihoods of farmers and their communities.

## Methods

### Study sites

**Fig. 1:**
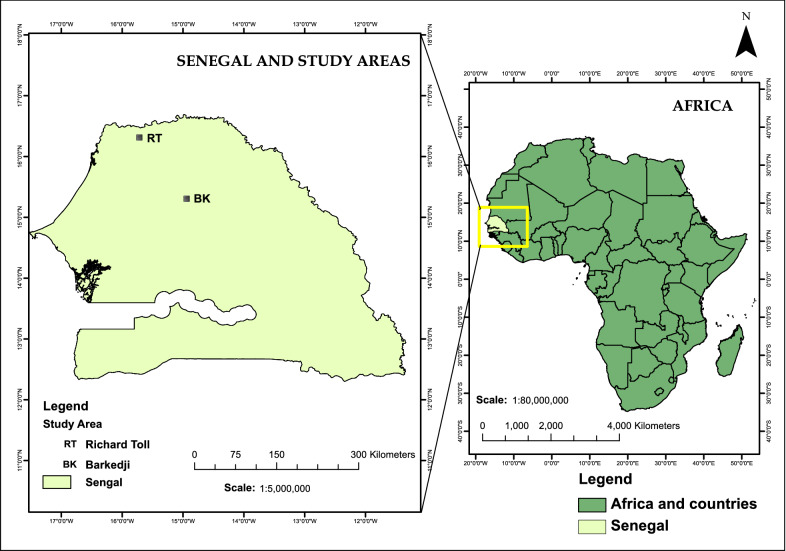
Map of the two study sites

This research was carried out in two regions in northern Senegal. Six villages were selected around the town of Barkedji (15.2774° N, 14.8674° W) in the Linguere department of the Louga region in the Vallée du Ferlo, and six villages around the Lac de Guiers (16.2247° N, 15.8408° W) near the town of Richard Toll in the Saint-Louis region in the Senegal River Basin (Fig. 1). The Richard Toll/Lac de Guiers area has undergone significant modifications such as desalination and the creation of irrigation canals, with permanent changes to local ecology, favoring expansion of snail intermediate host habitats, and increased sharing of water contact points by communities with their animals. In Barkedji, temporary ponds are an important source of water for human populations and their animals. These ephemeral water sources disappear completely during the dry season, interrupting transmission of schistosomiasis and necessitating seasonal migration by a large proportion of livestock-keeping communities. In both study areas, water contact points are used simultaneously by people and their livestock, encouraging the transmission of schistosomiasis between and within humans and animals [9]. In the area of Lac de Guiers, human schistosomiasis prevalence in humans can be as high as 88%, and 47% in Barkedji [9]. In Senegal, *S. bovis*, *S. curassoni* and hybrids of *S. bovis*:*S. curassoni* are the prevalent species causing livestock schistosomiasis [6, 12]. Recent work of Léger et al. [9] on livestock schistosomiasis revealed that *S. bovis* is the primary species causing livestock schistosomiasis in the Lac de Guiers area and *S. curassoni* in the Barkedji area. The prevalence estimates in slaughtered livestock in the two regions were as high as 85% for Lac de Guiers and 92% for Barkedji [9].

### Study overview

First, a generic partial budget model for the estimation of disease costs was conceptualised and data needs identified based on knowledge of the effects of livestock schistosomiasis and variables commonly used in impact studies of livestock disease. Subsequently, protocols were developed for a cross-sectional interview-based survey and focus group discussions (FGDs) with farmers covering questions on knowledge, occurrence and manifestations of livestock schistosomiasis, herd and production data, and management of livestock and disease.

The data collected were analysed and used to develop and parameterise specific production and partial budget models for the two sites and to define scenarios in line with local production and management practices. Secondary data and expert opinion were collated to complement the primary data where needed. Finally, livestock schistosomiasis disease costs were estimated for herds or flocks of cattle, sheep and goats using stochastic simulations in RiskAMP Add-in software for Excel with 10,000 iterations for a time frame of 1 year.

### Primary data collection and use

#### Participant selection

Target participants were subsistence and transhumance livestock farmers, i.e., the predominant ruminant production system in the two regions, rearing cattle, sheep and/or goats whose livestock products are consumed by the farmers’ households or sold to neighbours/at the local market. The selling of animals often takes place on a need basis to cover expenditures such as school fees; if there is no need, assets are commonly stored in the form of a herd or flock.

#### Data collection and analysis

Of the 12 villages selected from Barkedji and the Lac de Guiers regions, eight had previously participated in the Zoonoses and Emerging Livestock Systems (ZELS) project, and four villages (two in each region) were newly recruited. For the cross-sectional survey, questions were encoded in Open Data Kit (ODK) mobile data collection software. The questionnaire covered the following topics: demographics, production and management practices (including disease management and selling of animals and products), impact of livestock deaths on livelihood, prevention behaviour in people and animals, knowledge of disease in humans and livestock, signs of the disease in livestock, and equity. Most questions were closed, while a few were open. The full survey questionnaire is available upon request from the corresponding author(s). Each survey participant was also asked to complete a table about the number of animals owned per species, age group (young, adult), sex and breed (local, exotic or cross-bred); this information can be found in Additional file 1. The survey was translated from English to French and administered by local enumerators following a training session with the researchers leading the fieldwork.

Farmers who participated in the survey were also invited to participate in FGDs and participatory group activities to gather data on general signs of animal disease, signs of schistosomiasis in livestock, selling and buying of animals, milk and meat, feed and medicine including prices. All group activities were facilitated by a local enumerator with one person acting as note taker; the language used was Wolof. The full question guide can be found in Additional file 2. Summary notes were generated, and the discussions were recorded in full. The recordings were transcribed and then translated into English by the Senegalese research collaborators.

Data were collected in August and September 2019. Upon completion of the survey, data were downloaded from ODK and stored as an Excel file on a safe Royal Veterinary College [University of London] (RVC) drive. The tables on livestock numbers were collected as hard copies and manually added to the Excel file using the identifier code given to each participant. The translated transcripts of the FGDs were sent to the research team based at the RVC for storage and analysis.

### Consent and ethical approval

For all primary data collection activities, the researchers first explained what the study was about, how the data collection would work and the rights of the participants. Following that, each participant was asked to give their consent, which was either recorded as oral or written consent in the survey software or as written consent for the FGDs. Ethical approval was sought and granted by the (i) Clinical Research and Ethical Review Board at the RVC, approval numbers URN 20151327 and 2019 1899-3; and (ii) the Comité National d’Ethique pour la Recherche en Santé (Dakar, Senegal), approval numbers SEN15/68 and SEN 19/68.

### Data cleaning and analysis

Survey data were checked for completeness and cleaned, which entailed mainly harmonisation of spelling in open question fields. Answers available in French in the open comment fields were translated to English by the authors and professional translators. Data on the demographics of respondents, knowledge on schistosomiasis and the economic impact of the disease were analysed. Microsoft Excel was used to calculate summary statistics and to visualise the data. For uncertain variables (e.g. those with skewed distributions, inconsistency or too few responses), probability distributions were assigned. The open questions were read in detail in the search for information that would be relevant for the conceptualisation of the economic models including the definition of scenarios; relevant information was extracted as summary statements. For example, some respondents stated that sick animals in the herd will lose value and condition and explained a need to replace them with new ones; this informed the replacement strategy used in building the models. Data about why livestock are kept, milking animals with schistosomiasis, and which animals are sold and bought were extracted from individual interviews. Data from the group activities were analysed to identify information on daily feed quantity and type of feed consumed by animals, cost of feed, whether or not farmers sell sick animals, and questions on whether animals with schistosomiasis sell differently. Common topics were identified across responses for the FGDs and interviews which were used to inform the structure of the partial budget model and the input variables.

### Estimation of the financial impact of livestock schistosomiasis

#### Model development and scenarios

Stochastic models were developed in Microsoft Excel with the RiskAMP Add-in for simulation modelling; they are available on request from the corresponding author. Programme evaluation and review technique (PERT) distribution was assigned to the identified uncertain parameters. The information gained from the analysis of the primary data collected, available literature and expert opinion was used to decide on what species to include, and to define scenarios for the financial impact analysis. The data were used to model a representative herd or flock for each species including the number of animals per age group and sex. Further, the information was used to define scenarios for the analysis.

Integrated production and partial budget analysis models were set up for 1 year, which is approximately the production cycle of lactating cows in the study populations. Two scenarios were considered based on the most common practices reported by respondents. Scenario 1 was a situation where farmers would test and treat their animals when seeing clinical signs consistent with livestock schistosomiasis. Scenario 2 was a situation where farmers would not test or treat their animals when seeing schistosomiasis in their herds or flocks. Detailed scenario descriptions are given in Table 1.

**Table 1:** Definitions of scenarios for the partial budget analysis. Scenario-specific input parameters are given in Table 3

	Scenario 1: Farmers who consult veterinarians and test for schistosomiasis in their animals	Scenario 2: Farmers who do not consult veterinarians or test or treat their animals	Reasoning	Information source for reasoning
Testing strategy	A defined proportion of sick^a^ animals will be tested	No sick animal is tested	Not all farmers test the animals. Those who have health-seeking behaviour might not be able to afford the cost of testing of sick animals	Primary data: Survey
Treatment strategy	A defined proportion of tested animals will be treated and a defined proportion of untested animals will be treated	No sick animals is treated for schistosomiasis	Not all farmers who test can afford the treatment costs for all the animals. Not all farmers can afford the treatment costs for all sick animals	Primary data: Survey
Effectiveness of treatment	The sick animals that are treated with praziquantel recover following the treatment	Not applicable	Praziquantel is the medical treatment most commonly used and it is known to be effective	Primary data: Survey; literature [35]
Replacement strategy	Treated animals will recover and not be replaced. The majority of untreated sick animals, irrespective of age, will be sold at a lower market price. A proportion of the animals sold will be replaced with the same type of animal (young for young, adult for adult)	The majority of sick animals, irrespective of age, will be sold at a lower market price. A proportion of the animals sold will be replaced with the same type of animal (young for young, adult for adult)	Sick animals in the herd will lose value and condition, hence the need to replace them with new ones	Primary data: Group discussion
Feed and supplement quantity	No change in feed and supplement quantity for sick animals	No change in feed and supplement quantity for sick animals	There will not be an increase in feed quantity for sick animals, but they will lose condition, because of the higher energy requirement	Primary data: Group discussion and survey; expert opinion
Milk yield and lactation duration	Sick animals will have reduced milk yield and a shorter lactation period compared to healthy females	Sick animals will have reduced milk yield and a shorter lactation period compared to healthy females	Animals that are sick because of schistosomiasis have a lower milk yield and a shorter lactation period	Literature: [33]

^a^Sick animals are animals with clinical signs

#### Partial budget analysis

The financial impact per year was the net value estimated for each species and scenario using the following basic equation:

1 $${\text{Net value }} = \, \left( {{\text{Costs saved }} + {\text{ Added revenue}}} \right) \, {-} \, \left( {{\text{New costs }} + {\text{ Revenue forgone}}} \right)$$

Each of the six models (two scenarios per species, three species in total) had distinct input parameters as listed in Table 2 (general input variables) and Table 3 (scenario-specific input variables).

**Table 2:** General input variables used to estimate disease costs (animal numbers, production parameters, morbidity rates and prices)

Variable	Unit	Notation	Value for cattle	Value for sheep	Value for goats	Explanation	References
Proportion of lactating females among adult animals	%	*P*_LF_	0.53	0.63	0.63	Estimated based on survey data considering the ratio of dry to lactating animals and the median proportion of female animals in a herd	Survey
Number of young animals	Heads	*N*_Y_	6	26	26	Information provided by the respondents	Survey
Number of adult animals	Heads	*N*_A_	16	35	35		
Morbidity rate in young animals	Year^−1^	Mb_Y_	Pert (0.017, 0.021, 0.025)	Pert (0.1, 0.125, 0.15)	Pert (0.1, 0.125, 0.15)	High morbidity rate due to the reported high prevalence of schistosomiasis in the regions	Expert opinion and literature
Morbidity rate in adult animals	Year^−1^	Mb_A_	Pert (0.017, 0.021, 0.025)	Pert (0.1, 0.125, 0.15)	Pert (0.1, 0.125, 0.15)		
Average duration of clinical illness if animal treated (days)	d	*D*_CIT_	7.00	7.00	7.00	When praziquantel is used, the animals will improve within a few days, as parasites start dying very soon	Assumption
Average duration of clinical illness if animal is not treated (days)	d	*D*_CI_	183.0	183.0	183.0	When animals are not treated, they will not recover and will be continually ill. Infection and clinical illness could start at the beginning of the year or anytime throughout. Here, a mid-year infection and subsequent clinical illness is assumed	Assumption
Average duration of lactation in healthy females	d	*D*_LF_	270.0	260.0	260.0	In cows, average duration for lactation in Senegal is 210–270 days (7 to 9 months); the majority of respondents reported a lactation duration of 6 to 12 months. Therefore, the 9-month value was chosen. In dams, average duration for lactation is 260 days according to existing literature	Literature [32, 36, 37] and survey
Average duration without the animals sold and not replaced in the herd/flock	d	*D*_S_	183.0	183.0	183.0	It is assumed that animals are sold mid-year and will therefore not be present in the herd or flock for half of the year	Assumption
Daily milk quantity in healthy female	l	*M*_HA_	Pert (1.0, 2, 3.5)	Pert (0.5, 1.0, 1.2)	Pert (0.5, 1.0, 1.2)	Median values from survey used as a basis	Survey
Daily concentrate feed quantity in healthy animals	kg	*F*_HA_	Pert (3.0, 4.0, 5.0)	Pert (0.8, 1, 1.2)	Pert (0.8, 1, 1.2)	Value mentioned most often in group discussion	FGD
Daily supplement quantity in healthy animals	kg	*S*_HA_	Pert (0.8, 0.1, 0.12)	Pert (0, 0.025, 0.04)	Pert (0, 0.025, 0.04)	Value from literature	Literature
Market price for young healthy animal	XOF	Pr_YHA_	Pert (216000, 270000, 324000)	Pert (29000, 36250, 43500)	26,250	Information provided by the respondents	FGD
Market price for young sick animal	XOF	Pr_YSA_	Pert (160000, 200000, 240000)	Pert (21460, 26825, 32190)	20,000		
Market price for adult healthy animal	XOF	Pr_AHA_	Pert (304000, 380000, 456000)	Pert (32000, 40000, 48000)	38,000		
Market price for adult sick animal	XOF	Pr_ASA_	Pert (264000, 330000, 396000)	Pert (27840, 34800, 41760)	30,000		
Price of milk per litre for healthy animals	XOF	Pr_MHA_	557.92	601.70	530.88		
Price of milk per litre for sick animals	XOF	Pr_MSA_	525.00	500.00	500.00	Information provided by the respondents and triangulated with survey data from [38]	FGD and [38]
Price of concentrate feed per kg	XOF	Pr_F_	Pert (34.6, 43, 51.9)	Pert (34.6, 43, 51.9)	Pert (34.6, 43, 51.9)	Price of feed as reported by respondents	Survey
Price of supplement per kg	XOF	Pr_S_	452.00	452.00	452.00	Calculated based on data from [38]	Literature [38]
Price of testing per animal	XOF	Pr_Te_	1050.00	Pert (75.24, 83.6, 91.96)	Pert (75.24, 83.6, 91.96)	Information provided by the respondents	Survey, FGD
Price of routine treatment per animal per day	XOF	Pr_RT_	Pert (18, 23, 28)	Pert (18, 23, 28)	Pert (18, 23, 28)	Medical expenditure for animals in a herd include e.g. vaccination, deworming, tick treatment	Survey and [38]
Price of clinical treatment per animal (for veterinary-use praziquantel)	XOF	Pr_Tr_	Pert (510.35, 567.05, 623.76)	Pert (510.35, 567.05, 623.76)	Pert (510.35, 567.05, 623.76)	Price of a praziquantel tablet for animals that have a clinical disease caused by schistosomiasis	Literature [39]

**Table 3:** Scenario-specific input variables used to estimate disease costs (schistosomiasis-related disease effects and the reaction to the disease)

Variable	Unit	Notation	Cattle		Sheep and goats		Explanation	References
			Scenario 1	Scenario 2	Scenario 1	Scenario 2		
Average duration of clinical illness before animal is sold	d	*D*_CIS_	14.0	7.0	14.0	7.0	Number of days animals stay in the herd/flock before being sold; this reflects the observation and decision time of the farmer. It is assumed that they will observe the animal to see whether it recovers and then sell it. It is also assumed that those sold are sold early to get a better market price, when they still have some condition	Assumption
Proportion of sick animals tested	%	*P*_TS_	Pert (0.24, 0.31, 0.36)	0.00	Pert (0.24, 0.3, 0.36)	0.00	Only a handful of the animals showing clinical signs will be tested. Those who have health-seeking behaviour might not be able to afford the cost of testing for all sick animals	Assumption based on literature [13]
Proportion of tested animals that are treated	%	*P*_TT_	Pert (0.40, 0.50, 0.60)	0.00	Pert (0.40, 0.50, 0.60)	0.00	Not all farmers who test will be able to afford the treatment costs for all the animals; hence only some will treat	Assumption
Proportion of untested animals that are treated	%	*P*_UTT_	Pert (0.64, 0.80, 0.96)	0.00	Pert (0.64, 0.80, 0.96)	0.00	It is assumed that farmers with health-seeking behaviour will treat some of the sick animals	Assumption
Proportion of sick animals sold among those not treated	%	*P*_SUT_	Pert (0.90, 0.95, 1.00)	Pert (0.90, 0.95, 1.00)	Pert (0.90, 0.95, 1.00)	Pert (0.90, 0.95, 1.00)	Farmers reported in the survey that they sell all types of animals (young, adult, old, production and breeding animals). Many also indicated selling animals when they are sick. It is assumed that farmers will sell both the treated and untreated sick animals	Survey and assumption
Proportion of sick animals sold that are replaced	%	*P*_SAR_	Pert (0.56, 0.70, 0.84)	Pert (0.40, 0.50, 0.60)	Pert (0.56, 0.70, 0.84)	Pert (0.40, 0.50, 0.60)	Because farmers like to maintain their herds (their asset), it is assumed that a proportion of the animals sold will be replaced. Because farmers in scenario 2 have more animals to sell, their replacement rate is lower, as they will not have the means to replace so many animals	Assumption
Rate of reduced lactation duration in sick females (due to disease)	Year^−1^	*R*_LF_	Pert (0.032, 0.04, 0.048)	Pert (0.032, 0.04, 0.048)	Pert (0.10, 0.12, 0.15)	Pert (0.10, 0.12, 0.15)	The lactation duration of sick females will be shortened	Assumption based on literature [13, 40]
Rate of reduced milk yield in sick females (due to disease)	Year^−1^	*R*_MY_	Pert (0.08, 0.1, 0.12)	Pert (0.08, 0.10, 0.12)	Pert (0.08, 0.10, 0.12)	Pert (0.08, 0.10, 0.12)	The milk yield of sick females will be reduced	Survey, FGD
Mortality rate young animal among those sick and not sold	Year^−1^	Mt_Y_	Pert (0.032, 0.04, 0.048)	Pert (0.032, 0.04, 0.048)	Pert (0.40, 0.50, 0.60)	Pert (0.40, 0.50, 0.60)	Information by respondents and expert opinion. Mortality due to schistosomiasis in cattle/sheep/goats is low in regular production years	Survey, expert opinion
Mortality rate adult animal among those sick and not sold	Year^−1^	Mt_A_	Pert (0.032, 0.04, 0.048)	Pert (0.032, 0.04, 0.048)	Pert (0.40, 0.50, 0.60)	Pert (0.40, 0.50, 0.60)		

Scenario 1 relates to farmers who consult veterinarians and test for schistosomiasis in their animals; scenario 2 relates to farmers who do not consult veterinarians or test or treat their animals

New costs were additional costs for testing and treatment and replacement of sick animals.

For scenario 1, this included the following costs:

2 $${\text{Testing of young sick animals }} = \, N_{\text{Y}} *{\text{ Mb}}_{\text{Y}} * \, P_{{{\text{TS}}}} *{\text{ Pr}}_{{{\text{Te}}}},$$

where *N*_Y_ stands for the number of young animals, Mb_Y_ the morbidity rate of young animals, *P*_TS_ the proportion of sick animals tested, and Pr_Te_ the price of testing per animal.

3 $${\text{Testing of adult sick animals }} = \, N_{\text{A}} *{\text{ Mb}}_{\text{A}} * \, P_{{{\text{TS}}}} *^{{}} {\text{Pr}}_{{{\text{Te}}}},$$

where *N*_A_ stands for the number of adult animals, and Mb_A_ the morbidity rate of adult animals.

4 $${\text{Treatment for sick animals tested }} = \, \left( {N_{\text{A}} *{\text{ Mb}}_{\text{A}} + \, N_{\text{Y}} *{\text{ Mb}}_{Y} } \right) \, * \, P_{{{\text{TS}}}} * \, P_{{{\text{TT}}}}^{{}} *{\text{ Pr}}_{{{\text{Tr}}}},$$

where *P*_TT_ stands for the proportion of tested animals that are treated, and Pr_Tr_ the price of clinical treatment per animal.

5 $${\text{Treatment for sick animals not tested }} = \, \left( {N_{\text{A}} *{\text{ Mb}}_{A} + \, N_{\text{Y}} *{\text{ Mb}}_{\text{Y}} } \right) \, *^{{}} \left( {1 \, - \, P_{{{\text{TS}}}} } \right) \, * \, P_{{{\text{UTT}}}}^{{}} *{\text{ Pr}}_{{{\text{Tr}}}},$$

where *P*_UTT_ stands for the proportion of untested animals treated.

For scenarios 1 and 2, this included the following costs:

6 $$\begin{aligned}& {\text{Replacing sick animals sold }} = \, (N_{\text{A}} *{\text{ Mb}}_{\text{A}} *{\text{Pr}}_{{{\text{AHA}}}} + \, N_{\text{Y}} *{\text{ Mb}}_{\text{Y}} *{\text{ Pr}}_{{{\text{YHA}}}} ) \, \hfill \\ &\quad *^{{}} [P_{{{\text{TS}}}} * \, (1 \, - P_{{{\text{TT}}}} ) \, + \, \left( {1 \, - \, P_{{{\text{TS}}}} } \right) \, * \, \left( {1 - \, P_{{{\text{UTT}}}} } \right)] \, * \, P_{{{\text{SUT}}}} * \, P_{{{\text{SAR}}}}, \hfill \\ \end{aligned}$$

where Pr_AHA_ stands for the market price of an adult healthy animal, Pr_YHA_ the market price of a young healthy animal, *P*_SUT_ the proportion of sick animals sold among those not treated, and *P*_SAR_ the proportion of young sick animals sold that are replaced.

Revenue forgone stemmed from milk not sold or sold at a lower price and selling animals at a lower market value. For scenarios 1 and 2, this included revenue forgone as follows:

7 $$\begin{gathered} {\text{Milk not sold from sick females }}\left( {\text{kept in the herd}} \right){\text{ due to shortened lactation }} = \, N_{\text{A}} *{\text{ Mb}}_{\text{A}} * \, P_{{{\text{LF}}}} * \hfill \\ \quad \, \left[ {P_{{{\text{TS}}}} * \, \left( {1 \, - \, P_{{{\text{TT}}}} } \right) \, + \, \left( {1 \, - \, P_{{{\text{TS}}}} } \right) \, * \, \left( {1 \, - \, P_{{{\text{UTT}}}} } \right)} \right] \, * \, \left( {1 \, - \, P_{{{\text{SUT}}}} } \right) \, *\left( {1 - \, R_{{{\text{LF}}}} } \right)* D_{{{\text{CI}}}} *R_{{{\text{MY}}}} *M_{{{\text{HA}}}} *{\text{Pr}}_{{{\text{MHA}}}}, \hfill \\ \end{gathered}$$

where *P*_LF_ stands for the proportion of lactating females among the adult animals, *R*_LF_ the rate of reduced lactation duration in sick females, *D*_CI_ the duration of clinical illness if an animal is not treated, *R*_MY_ is the rate of reduced milk yield in sick females, *M*_HA_ the daily milk quantity in healthy animals, and Pr_MHA_ the price of milk per litre for a healthy animal.

8 $$\begin{aligned} & {\text{Milk not sold from sick females }}\left( {\text{kept in the herd}} \right){\text{ due to reduced milk production per day }} \\ & \quad = & \, N_{\text{A}} *{\text{ Mb}}_{\text{A}} * \, P_{{{\text{LF}}}} * \, \left[ {P_{{{\text{TS}}}} * \, \left( {1 \, - \, P_{{{\text{TT}}}} } \right) \, + \, \left( {1 \, - \, P_{{{\text{TS}}}} } \right) \, * \, \left( {1 \, - \, P_{{{\text{UTT}}}} } \right)} \right] \, \\& * \, \left( {1 \, - \, P_{{{\text{SUT}}}} } \right) \, * \, D_{{{\text{CI}}}} * \, R_{{{\text{MY}}}} *M_{{{\text{HA}}}} *{\text{ Pr}}_{{{\text{MHA}}}} \\ \end{aligned}$$

9 $$\begin{gathered} {\text{Milk sold from sick females }}\left( {\text{kept in the herd}} \right){\text{ at lower market price}} \hfill \\ \quad = \, N_{\text{A}} *{\text{ Mb}}_{\text{A}} * \, P_{{{\text{LF}}}} * \, \left[ {P_{{{\text{TS}}}} * \, \left( {1 \, - \, P_{{{\text{TT}}}} } \right) \, + \, \left( {1 \, - \, P_{{{\text{TS}}}} } \right) \, * \, \left( {1 \, - \, P_{{{\text{UTT}}}} } \right)} \right] \, \hfill \\ \quad \;* \, \left( {1 \, - \, P_{{{\text{SUT}}}} } \right) \, * \, D_{{{\text{CI}}}} * \, R_{{{\text{MY}}}} *M_{{{\text{HA}}}} * \, ({\text{Pr}}_{{{\text{MHA}}}} - {\text{Pr}}_{{{\text{MSA}}}} ), \hfill \\ \end{gathered}$$

where Pr_MSA_ is the price of milk per litre for a sick animal.

10 $$\begin{aligned}& {\text{Milk sold from sick females }}\left( {\text{before the sick females are sold}} \right){\text{ at lower market price}} \hfill \\ &\, = N_{\text{A}} *{\text{ Mb}}_{\text{A}} * \, P_{{{\text{LF}}}} * \, \left[ {P_{{{\text{TS}}}} * \, \left( {1 \, - \, P_{{{\text{TT}}}} } \right) \, + \, \left( {1 \, - \, P_{{{\text{TS}}}} } \right) \, * \, \left( {1 \, - \, P_{{{\text{UTT}}}} } \right)} \right] \, \hfill \\ & \quad * \, P_{{{\text{SUT}}}} *D_{{{\text{CIS}}}} * \, R_{{{\text{MY}}}} *M_{{{\text{HA}}}} * \, ({\text{Pr}}_{{{\text{MHA}}}} - {\text{Pr}}_{{{\text{MSA}}}} ), \hfill \\ \end{aligned}$$

where *D*_CIS_ is the average duration of clinical illness before the animal is sold.

11 $$\begin{aligned}& {\text{Sick animals sold at lower market price }} \\ &\quad = \, \left[ {N_{\text{A}} *{\text{ Mb}}_{\text{A}} * \, \left( {{\text{Pr}}_{{{\text{AHA}}}} - {\text{ Pr}}_{{{\text{ASA}}}} } \right) \, + \, N_{\text{Y}} *^{{}} {\text{Mb}}_{\text{Y}} * \, \left( {{\text{Pr}}_{{{\text{YHA}}}} - {\text{ Pr}}_{{{\text{YSA}}}} } \right)} \right] \, \\ & \quad \quad * \, \left[ {P_{{{\text{TS}}}} * \, \left( {1 \, - \, P_{{{\text{TT}}}} } \right) \, + \, \left( {1 \, - \, P_{{{\text{TS}}}} } \right) \, * \, \left( {1 \, - \, P_{{{\text{UTT}}}} } \right)} \right] \, * \, P_{{{\text{SUT}}}}, \\ \end{aligned}$$

where Pr_ASA_ stands for the market price of an adult sick animal and Pr_YSA_ for the market price of a young sick animal.

12 $$\begin{aligned} &{\text{Value reduction of sick animals not sold }}\left( {\text{but alive}} \right) \, \hfill \\ &\quad = \left[ {N_{\text{A}} *{\text{ Mb}}_{\text{A}} * \, \left( {{\text{Pr}}_{{{\text{AHA}}}} - {\text{ Pr}}_{{{\text{ASA}}}} } \right) \, * \, \left( {1 \, - {\text{ Mt}}_{\text{A}} } \right) \, + \, N_{\text{Y}} *{\text{ Mb}}_{\text{Y}} * \, \left( {{\text{Pr}}_{{{\text{YHA}}}} - {\text{ Pr}}_{{{\text{YSA}}}} } \right)} \right] \, \hfill \\& \quad \quad * \, \left( {1 \, - {\text{ Mt}}_{Y} } \right) \, * \, \left[ {P_{{{\text{TS}}}} * \, \left( {1 \, - \, P_{{{\text{TT}}}} } \right) \, + \, \left( {1 \, - \, P_{{{\text{TS}}}} } \right) \, * \, \left( {1 \, - \, P_{{{\text{UTT}}}} } \right)} \right] \, * \, (1 \, - \, P_{{{\text{SUT}}}} ), \hfill \\ \end{aligned}$$

where Mt_A_ and Mt_Y_ are the mortality rates for adult and young animals, respectively, among those sick and not sold.

13 $$\begin{aligned} &{\text{Herd value reduction due to sick animals sold and not replaced }} \hfill \\ & \quad = \, (N_{\text{A}} *^{{}} {\text{Mb}}_{\text{A}} *{\text{ Pr}}_{{{\text{AHA}}}} + \, N_{\text{Y}} *^{{}} {\text{Mb}}_{\text{Y}} *{\text{ Pr}}_{\text{YHA}} ) \, \hfill \\ & \quad \quad * \, \left[ {P_{{{\text{TS}}}} * \, \left( {1 \, - \, P_{{{\text{TT}}}} } \right) \, + \, \left( {1 \, - \, P_{{{\text{TS}}}} } \right) \, * \, \left( {1 \, - \, P_{{{\text{UTT}}}} } \right)} \right] \, * \, P_{{{\text{SUT}}}} * \, (1 \, - \, P_{{{\text{SAR}}}} ) \hfill \\ \end{aligned}$$

14 $$\begin{aligned}& {\text{Value reduction of sick, untreated animals not sold and dead }} \hfill \\& \quad = \, \left( {N_{\text{A}} *^{{}} {\text{Mb}}_{\text{A}} *{\text{ Pr}}_{{{\text{AHA}}}} *{\text{ Mt}}_{\text{A}} + \, N_{\text{Y}} *^{{}} {\text{Mb}}_{\text{Y}} *{\text{ Pr}}_{{{\text{YHA}}}} *{\text{ Mt}}_{\text{Y}} } \right) \hfill \\ &\, \quad * \, \left[ {P_{{{\text{TS}}}} * \, \left( {1 \, - \, P_{{{\text{TT}}}} } \right) \, + \, \left( {1 \, - \, P_{{{\text{TS}}}} } \right) \, * \, \left( {1 \, - \, P_{{{\text{UTT}}}} } \right)} \right] \, * \, \left( {1 \, - \, P_{{{\text{SUT}}}} } \right) \hfill \\ \end{aligned}$$

Expenditures saved stemmed from saving concentrate feed, supplements and routine treatment. For scenarios 1 and 2, this included expenditures saved from the following:

15 $$\begin{aligned}& {\text{Concentrate feed saved on sick animals sold and not replaced }} \hfill \\& \quad = \, \left( {N_{\text{A}} *{\text{ Mb}}_{\text{A}} * + \, N_{\text{Y}} *{\text{ Mb}}_{\text{Y}} } \right) \, *^{{}} [P_{{{\text{TS}}}} * \, (1 \, - P_{{{\text{TT}}}} ) \, + \, \left( {1 \, - \, P_{{{\text{TS}}}} } \right) \, * \, \left( {1 \, - \, P_{{{\text{UTT}}}} } \right)] \, \hfill \\ & \quad \quad * \, P_{{{\text{SUT}}}} * \, \left( {1 \, - \, P_{{{\text{SAR}}}} } \right) \, * \, D_{S} *F_{{{\text{HA}}}} *{\text{ Pr}}_{{\text{F}}}, \hfill \\ \end{aligned}$$

where *D*_*S*_ stands for the average duration without the animals sold and not replaced in the herd/flock, *F*_HA_ the daily concentrate feed quantity in kilograms in healthy animals, and Pr_F_ the price of concentrate feed per kilogram.

16 $$\begin{aligned}& {\text{Concentrate feed saved on sick, untreated animals not sold and dead}} \hfill \\& \, = \, \left( {N_{\text{A}} *^{{}} {\text{Mb}}_{\text{A}} *{\text{ Mt}}_{A} + \, N_{\text{Y}} *^{{}} {\text{Mb}}_{\text{Y}} *{\text{ Mt}}_{\text{Y}} } \right) \, * \, \left[ {P_{{{\text{TS}}}} * \, \left( {1 \, - \, P_{{{\text{TT}}}} } \right) \, + \, \left( {1 \, - \, P_{{{\text{TS}}}} } \right) \, * \, \left( {1 \, - \, P_{{{\text{UTT}}}} } \right)} \right] \, \hfill \\& \quad * \, \left( {1 \, - \, P_{{{\text{SUT}}}} } \right) \, * \, D_{\text{S}} *F_{{{\text{HA}}}} *{\text{ Pr}}_{\text{F}} \hfill \\ \end{aligned}$$

17 $$\begin{aligned} & {\text{Supplement saved on sick animals sold and not replaced}} \hfill \\ &\, = \, \left( {N_{\text{A}} *{\text{ Mb}}_{\text{A}} * \, + \, N_{\text{Y}} *{\text{ Mb}}_{\text{Y}} } \right) \, *^{{}} [P_{{{\text{TS}}}} * \, (1 \, - P_{{{\text{TT}}}} ) \, + \, \left( {1 \, - \, P_{{{\text{TS}}}} } \right) \, * \, \left( {1 \, - \, P_{{{\text{UTT}}}} } \right)] \, \hfill \\ & \quad * \, P_{{{\text{SUT}}}} * \, \left( {1 \, - \, P_{{{\text{SAR}}}} } \right) \, * \, D_{\text{S}} *S_{{{\text{HA}}}} * \, \text{Pr}_{{{\text{Su}}}}, \hfill \\ \end{aligned}$$

where *S*_HA_ stands for daily supplement quantity in kilograms in healthy animals and Pr_Su_ the supplement price per kilogram.

18 $$\begin{aligned} & {\text{Supplement saved on sick, untreated animals not sold and dead}} \hfill \\& \, = \, \left( {N_{\text{A}} *^{{}} {\text{Mb}}_{\text{A}} *{\text{ Mt}}_{\text{A}} + \, N_{\text{Y}} *^{{}} {\text{Mb}}_{\text{Y}} *{\text{ Mt}}_{\text{Y}} } \right) \, * \, \left[ {P_{\text{TS}} * \, \left( {1 \, - \, P_{{{\text{TT}}}} } \right) \, + \, \left( {1 \, - \, P_{{{\text{TS}}}} } \right) \, * \, \left( {1 \, - \, P_{{{\text{UTT}}}} } \right)} \right] \, \hfill \\& \quad * \, \left( {1 \, - \, P_{{{\text{SUT}}}} } \right) \, * \, D_{\text{S}} *S_{{{\text{HA}}}} *{\text{ Pr}}_{{{\text{Su}}}} \hfill \\ \end{aligned}$$

19 $$\begin{aligned}& {\text{Routine treatment saved on sick animals sold and not replaced}} \hfill \\& \, = \, \left( {N_{\text{A}} * \, \text{Mb}_{\text{A}} * \, + \, N_{\text{Y}} * \, \text{Mb}_{\text{Y}} } \right) \, *^{{}} [P_{\text{T}S} * \, (1 \, - P_{\text{TT}} ) \, + \, \left( {1 \, - \, P_{\text{TS}} } \right) \, * \, \left( {1 \, - \, P_{\text{UTT}} } \right)] \hfill \\& \quad \, * \, P_{\text{SUT}} * \, \left( {1 \, - \, P_{\text{SAR}} } \right) \, * \, D_{S} * \, \text{Pr}_{\text{RT}}, \hfill \\ \end{aligned}$$

where Pr_RT_ stands for the price of routine treatment per animal per day.

20 $$\begin{aligned}& {\text{Routine treatment saved on sick, untreated animals not sold and dead}} \hfill \\ &\, = \, \left( {N_{\text{A}} *^{{}} {\text{Mb}}_{\text{A}} *{\text{ Mt}}_{\text{A}} + \, N_{\text{Y}} *^{{}} {\text{Mb}}_{\text{Y}} *{\text{ Mt}}_{\text{Y}} } \right) \, * \, \left[ {P_{{{\text{TS}}}} * \, \left( {1 \, - \, P_{{{\text{TT}}}} } \right) \, + \, \left( {1 \, - \, P_{{{\text{TS}}}} } \right) \, * \, \left( {1 \, - \, P_{{{\text{UTT}}}} } \right)} \right] \, \hfill \\ & \quad * \, \left( {1 \, - \, P_{{{\text{SUT}}}} } \right) \, * \, D_{\text{S}} *{\text{Pr}}_{{{\text{RT}}}} \hfill \\ \end{aligned}$$

Extra revenue comprised the revenue from selling sick animals:

21 $$\begin{aligned} & {\text{Revenue from sick animals sold due to disease}} \hfill \\ \, &= \, \left( {N_{\text{A}} *^{{}} {\text{Mb}}_{\text{A}} *{\text{ Pr}}_{{{\text{ASA}}}} + \, N_{\text{Y}} *^{{}} {\text{Mb}}_{\text{Y}} *{\text{ Pr}}_{{{\text{YSA}}}} } \right) \, \hfill \\& \quad * \, \left[ {P_{{{\text{TS}}}} * \, \left( {1 \, - \, P_{{{\text{TT}}}} } \right) \, + \, \left( {1 \, - \, P_{{{\text{TS}}}} } \right) \, * \, \left( {1 \, - \, P_{{{\text{UTT}}}} } \right)} \right] \, * \, P_{{{\text{SUT}}}} \hfill \\ \end{aligned}$$

The partial budget models did not consider the effect on labour, as these production systems rely predominantly on unpaid family labour. All prices used for the models were in Senegalese currency, i.e., the West African CFA franc; 1 XOF = 0.0014 GBP as at the time of analysis (2020). Each partial budget analysis model was run with 10,000 iterations, and the net values were assigned as outputs. Finally, the impact of uncertain variables on the output of models (net value) was conducted using the built-in function performing univariate regression analysis.

## Results

### Respondent demographics

A total of 92 respondents representing different households participated in the survey; demographic characteristics are shown in Table 4.

**Table 4:** Demographic characteristics of survey respondents, *n* = 92

Characteristic	Number (percentage)
Gender	
Male	71 (77)
Female	21 (23)
Age	
Below 20 years	7 (8)
21–30 years	20 (22)
31–40 years	22 (24)
41–50 years	21 (23)
51–60 years	16 (17)
Above 60 years	6 (7)
Location	
Mayel (Barkedji)	11 (12)
Didjiery (Richard Toll)	9 (10)
Loumbel Mbada (Linguere)	9 (10)
Medina Cheikhou (Lac de Guiers)	8 (9)
Ndombo (Lac de Guiers)	8 (9)
Pathe Badio (Lac de Guiers)	8 (9)
Barkedji (Linguere)	8 (9)
Mbane (Lac de Guiers)	8 (9)
Loumbel Lana (Linguere)	7 (8)
Ngao (Linguere)	6 (7)
Ngassama (Linguere)	6 (7)
Mourseyni (Lac de Guiers)	4 (4)
Occupation	
Livestock merchant	66 (71)
Farmer	34 (37)
Merchant	25 (27)
Housewife	6 (7)
Student	3 (3)
Teacher	1 (1)
Health worker	1 (1)
Source of income	
Breeding	58 (63)
Trade	13 (14)
Agriculture	11 (12)
Livestock sales	2 (2)
Agriculture and breeding	1 (1)
Breeding and fishing	1 (1)
Dependent on parents	1 (1)
Fishing	1 (1)
Student	1 (1)
Teaching	1 (1)
Not mentioned	2 (2)

### Production and disease management

Local, cross and exotic breeds of all three species were kept in the two study areas (Additional file 3). In both study areas, the predominant breeds in all species were local breeds. Cattle were regarded by survey respondents as the most important livestock (49% of respondents), followed by sheep (27% of respondents) and then goats (5% of respondents). The animals were mostly kept for dual production purposes such as meat and breeding, dairy and breeding or meat and dairy, and the triple combination of meat, dairy and breeding (Additional file 4). In the predominant breed, i.e., local breed, cattle, sheep and goats were kept mostly for the triple purpose of meat, dairy and breeding (41%, 34% and 35%, respectively) and the dual purpose of dairy and breeding (30% for cattle, 22% for sheep and 15% for goats). With regard to the treatment of animals, 57/92 respondents (62%) stated that they routinely treated their animals. A total of 84/92 respondents (91%) stated that they routinely gave their animals supplements.

### Signs of schistosomiasis in animals and schistosomiasis-related management practices

A total of 81/92 respondents (88%) reported that they knew that animals could be infected with schistosomiasis, while 11/92 respondents (12%) reported not knowing. The most common signs of schistosomiasis reported by survey respondents for cattle, sheep and goats are displayed in Table 5. A total of 48/92 respondents (52%) reported that they would seek advice from local veterinary workers if they thought their livestock had schistosomiasis; 33/92 respondents (36%) had never tested their livestock in the past for schistosomiasis and 28/92 respondents (85%) used a veterinary clinic. With regard to treatment, 35/92 respondents (38%) stated that they had treated their livestock for schistosomiasis in the last 4 years, with 33/92 respondents (36%) using “Tenicure” (PZQ-levamisole combination) to treat.

**Table 5:** Signs of schistosomiasis as reported by respondents in the survey

Signs in cattle	Number (percentage)
	*n* = 81
Weight loss	52 (64)
Hollowing around eye	52 (64)
Diarrhoea	28 (35)
Weakness	20 (25)
Blood in urine	12 (15)
Blood in stool	10 (12)
Abortion	3 (4)
Dehydration	2 (2)
Don’t know	9 (11)

Signs in sheep	Number (percentage)
	*n* = 20
Hollowing around eye	13 (65)
Weight loss	11 (55)
Diarrhoea	11 (55)
Blood in urine	7 (35)
Blood in stool	4 (20)
Weakness	4(20)
Abortion	2 (10)
Dehydration	1 (5)
Don't know	2 (10)

Signs in goats	Number (percentage)
	*n* = 71
Weight loss	48 (68)
Hollowing around eye	45 (63)
Diarrhoea	21 (30)
Weakness	13 (18)
Blood in stool	9 (13)
Blood in urine	8 (11)
Abortion	2 (3)
Dehydration	1 (1)
Don't know	9 (13)

### Net disease value estimated using partial budget analysis

Results for livestock schistosomiasis costs per animal and year in the three species studied are shown in Tables 6, 7 and 8. For cattle (Table 6), the median net disease value for a standard cattle herd with 22 animals was −13,408 XOF (min −45,508; max +10,808) for scenario 1 and −49,296 XOF (min −141,972; max +32,246) for scenario 2. For sheep (Table 7), the median net disease value for a standard sheep flock with 61 animals was XOF −27,227 (min −82,423; max +16,483) for scenario 1 and −70,072 XOF (min −219,980; max +80,956) for scenario 2. For goats (Table 8), the median net disease value for a standard goat herd with 61 animals was −27,694 XOF (min −76,654; max +7048) for scenario 1 and −70,281 XOF (min −196,835; max +60,321) for scenario 2. In all models, the largest contribution to the total net value was caused by replacement of animals, herd value reduction and revenue from young sick animals sold due to disease.

**Table 6:** Livestock schistosomiasis disease costs in XOF for a common cattle herd in Senegal considering two scenarios^﻿b^

	Item	Scenario 1	Scenario 2
Costs			
New costs	Testing of young sick animals	44	–
	Testing of adult sick animals	109	–
	Treatment for sick animals tested	45	–
	Treatment for sick animals not tested	161	–
	Replacing sick animals sold	23,903	83,740
Revenue foregone	Milk not sold from sick females (kept in the herd) due to shortened lactation	161	285
	Milk not sold from sick females (kept in the herd) due to reduced milk production per day	168	297
	Milk sold from sick females (kept in the herd) at lower market price	10	18
	Milk sold from sick females (before the sick females are sold) at lower market price	12	28
	Sick animals sold at lower market price	6560	29,116
	Value reduction of sick animals not sold (but alive)	388	3763
	Herd value reduction because of the sick animals sold and NOT replaced	13,460	82,096
	Value reduction of sick, untreated animals not sold and dead	84	161
Total		45,105	199,503
Benefits			
Costs saved	Concentrate feed saved on sick animals sold and not replaced	1190	7255
	Concentrate feed saved on sick, untreated animals not sold and dead	8	14
	Supplement saved on sick animals sold and not replaced	–	–
	Supplement saved on sick, untreated animals not sold and dead	–	–
	Routine treatment saved on sick animals sold and not replaced	164	1000
	Routine treatment saved on sick, untreated animals not sold and dead	1	2
Extra revenue	Revenue from sick animals sold due to disease	30,803	136,720
Total benefits		32,166	144,992
Net disease costs	Mean	−13,729	−49,476
	Median	−13,408	−49,296
	Min	−45,508	−141,972
	Max	+10,808	+32,246

^b^Scenario 1 relates to farmers who consult veterinarians and test for schistosomiasis in their animals; scenario 2 relates to farmers who do not consult veterinarians or test or treat their animals

**Table 7:** Livestock schistosomiasis disease costs in XOF for a common sheep flock in Senegal considering two scenarios^b^

	Item	Scenario 1	Scenario 2
Costs			
New costs	Testing of young sick animals	100	–
	Testing of adult sick animals	132	–
	Treatment for sick animals tested	845	–
	Treatment for sick animals not tested	2411	–
	Replacing sick animals sold	54,527	148,649
Revenue foregone	Milk not sold from sick females (kept in the herd) due to shortened lactation	1091	3905
	Milk not sold from sick females (kept in the herd) due to reduced milk production per day	1247	4443
	Milk sold from sick females (kept in the herd) at lower market price	211	751
	Milk sold from sick females (before the sick females are sold) at lower market price	164	333
	Sick animals sold at lower market price	8744	29,587
	Value reduction of sick animals not sold (but alive)	442	12,046
	Herd value reduction because of the sick animals sold and NOT replaced	27,357	128,426
	Value reduction of sick, untreated animals not sold and dead	3928	11,827
Total costs		101,199	339,968
Benefits			
Costs saved	Concentrate feed saved on sick animals sold and not replaced	7021	32,962
	Concentrate feed saved on sick, untreated animals not sold and dead	1007	3044
	Supplement saved on sick animals sold and not replaced	1379	6474
	Supplement saved on sick, untreated animals not sold and dead	198	598
	Routine treatment saved on sick animals sold and not replaced	3369	15,816
	Routine treatment saved on sick, untreated animals not sold and dead	483	1461
Extra revenue	Revenue from sick animals sold due to disease	73,140	247,488
Total benefits		86,598	307,843
Net disease costs	Mean	−28,042	−69,894
	Median	−27,227	−70,072
	Min	−82,423	−219,980
	Max	+16,483	+80,956

^b^Scenario 1 relates to farmers who consult veterinarians and test for schistosomiasis in their animals; scenario 2 relates to farmers who do not consult veterinarians or test or treat their animals

**Table 8:** Livestock schistosomiasis disease costs in XOF for a common goat herd in Senegal considering two scenarios^b^

	Item	Scenario 1	Scenario 2
Costs			
New costs	Testing of young sick animals	72	–
	Testing of adult sick animals	108	–
	Treatment for sick animals tested	601	–
	Treatment for sick animals not tested	2275	–
	Replacing sick animals sold	47,246	109,949
Revenue foregone	Milk not sold from sick females (kept in the herd) due to shortened lactation	1373	2586
	Milk not sold from sick females (kept in the herd) due to reduced milk production per day	1541	2941
	Milk sold from sick females (kept in the herd) at lower market price	90	171
	Milk sold from sick females (before the sick females are sold) at lower market price	83	125
	Sick animals sold at lower market price	10,010	34,742
	Value reduction of sick animals not sold (but alive)	408	5252
	Herd value reduction because of the sick animals sold and NOT replaced	16,907	112,715
	Value reduction of sick, untreated animals not sold and dead	2690	6397
Total costs		83,405	274,878
Benefits			
Costs saved	Concentrate feed saved on sick animals sold and not replaced	3961	26,409
	Concentrate feed saved on sick, untreated animals not sold and dead	629	1495
	Supplement saved on sick animals sold and not replaced	1283	8551
	Supplement saved on sick, untreated animals not sold and dead	204	484
	Routine treatment saved on sick animals sold and not replaced	2083	13,888
	Routine treatment saved on sick, untreated animals not sold and dead	331	786
Extra revenue	Revenue from sick animals sold due to disease	54,144	187,922
Total benefits		62,634	239,535
Net disease costs	Mean	−28,282	−70,144
	Median	−27,694	−70,281
	Min	−76,654	−196,835
	Max	+7048	+ 60,321

^b^Scenario 1 relates to farmers who consult veterinarians and test for schistosomiasis in their animals; scenario 2 relates to farmers who do not consult veterinarians or test or treat their animals

Sensitivity analyses showed that the market prices for young and adult healthy and sick animals had the greatest impact on the net value for all species, with the highest regression coefficients for the market price for adult healthy animals (0.355 to 0.542) followed by the market price for adult sick animals (0.253 to 0.381), the market price for young healthy animals (0.039 to 0.180), the market price for young sick animals (0.016 to 0.099), the daily feed quantity, the rate of reduced feed intake and the rate of reduced lactation (regression coefficients between 0.01 and 0.03). The proportion of untested animals that are treated also had a noticeable influence on the net value in scenario 1, with regression coefficients of 0.092 for goats, 0.069 for sheep and 0.067 for cattle. The morbidity rate in adult animals had regression coefficients of 0.019 (scenario 1, goats), 0.013 (scenario 2, goats) and 0.011 (scenario 1, sheep); the morbidity rate in young animals in goats had a regression coefficient of 0.012. The variable ‘sick animals sold that are replaced’ had regression coefficients of 0.021 (scenario 1, goats) and 0.013 (scenario 1, sheep). The other uncertain variables all had regression coefficients < 0.01.

## Discussion

In this study the financial impact of livestock schistosomiasis on livestock keepers in two regions of Senegal was shown to be substantial, particularly in scenario 2, i.e., a situation where farmers do not test and treat animals. We observed that the median disease costs in a representative herd for the areas studied were between 0.23 and 1.22 of the average annual income in rural Senegal, with the disease costs highest in small ruminants (the average monthly income for people living in rural Senegal is 57,461 XOF [41]). Thus, having schistosomiasis in a herd will reduce the farmers' livelihood and, in some instances, potentially cause a situation where basic needs can no longer be covered.

The survey data showed that farmers consult a veterinarian or veterinary technician for their animals to be tested, although no information was available on the specific diagnostic test(s) used here by the veterinary technicians (considering the setting of these areas, it is very unlikely that advanced diagnostic tests such as molecular tests were used). Because of the existing practice of selling sick animals, the financial impact estimated was caused mainly by the selling and buying of animals and changes in herd value. With weight loss being a prominent sign of schistosomiasis infection reported by respondents, sick animals fetch a lower market price and cause replacement costs for the farmer. Consequently, farmers have an interest in selling sick, untreated animals as soon as possible to avoid a further reduction in market price. With the clinical signs reported including weight loss, hollowing around the eyes and diarrhoea, sick animals are likely recognised as such by potential buyers, and they will only pay the price for a sick animal.

The subsistence and transhumance farmers studied sell animals based only on needs and usually maintain their herd or flock size as a capital asset; thus, the reduction in herd value was modelled explicitly. In partial budget models for farming units where products are sold to make profits, the change in herd value is not commonly incorporated in a partial budget [42, 43]. However, in a setting where the herd or flock is not used as a means to make a profit but functions as a social and capital asset, the estimation of its change in value appears justified. Using the models described, the loss in herd value was a major cost to the farmers, caused mainly by a reduction in animals, as it was assumed that not all animals could be replaced. This was also reflected in the sensitivity analysis, where the market prices of animals were shown to have the greatest influence on the financial impact. Because farmers not testing and treating will have a larger number of sick animals (than those that test and treat), but most likely will not have the means to replace all the animals they are selling, the financial impact for them was highest. This indicates that testing and treating animals has the potential to reduce the financial impact of livestock schistosomiasis in these populations.

A previously published study on rural development and poverty reduction reported that most people in Senegal contribute 50% of their family labour to subsistence livestock farming, which accounts for a 23.8% share of their average income [44]. Many of the respondents from the two study areas examined here considered disease in their livestock as a large economic loss. As these farmers place great importance on their livestock, it is not surprising that some of the farmers would test as well as treat, although the cost of the diagnostic test (1050 XOF) is higher than the medication for the disease. The costs of schistosomiasis treatment (567 XOF) seem to be affordable, yet many farmers were not testing or treating their animals. Farmers who do not test and treat could experience a range of constraints and have other economic priorities. In a study on the attitudes of farmers regarding animal welfare, Kauppinen et al. [45] reported that most farmers considered their welfare and that of their animals as mutually dependent. Though the farmers are aware that their animals can be infected with schistosomiasis, they may not understand that treating the animals also confers protection on them by also potentially interrupting the zoonotic transmission of the disease from animals to humans and preventing hybridisation of species. Thus, further studies may need to look in more depth at the health-seeking behaviour and farmers’ motivation for disease control.

The availability of the human formulation of PZQ and the lack of accessibility to a suitably dosed veterinary formula of the drug means that farmers may use donated PZQ intended only for human use to treat their livestock [11]. Consequently, a systematic mis-dosing, and particular under-dosing, of the drug in the animals can be identified as one of the factors which have led to the reported high prevalence of livestock schistosomiasis in the regions examined [9]. This is a One Health concern, as the use and cross- or misuse of PZQ in animals have been reported to potentiate resistance and reduce efficacy of the drug [10, 14, 46–48]. The People’s Republic of China has already employed potential bovine vaccine development for zoonotic *S. japonicum* in some regions, in addition to controlled PZQ treatment of bovines, setting the pace for an integrated approach to schistosomiasis, simultaneously combining mitigation measures in animals with control measures in humans as part of its national control programme [49].

The multisectoral and inter-ministerial approach used in China leveraged technological advancements and socio-economic changes [50]. For example, one mitigation measure was to detect the intermediate host, *Oncomelania* snails, through DNA extraction and loop-mediated isothermal amplification (LAMP), and control the snails using mechanised tractor-plough molluscicide dispensers on marshland regions endemic for *S. japonicum* [51, 52]. In addition, treating bovines against schistosomiasis caused by *S. bovis* can interrupt the transmission of the disease from animals to humans by preventing possible environmental contamination by schistosomal eggs shed in the faeces of buffaloes [53, 54]. China’s prevalence of schistosomiasis in humans and bovines is now less than 1% [54]. If countries in Africa were to follow the Chinese example of integrated schistosomiasis control, the estimated high prevalence in humans and animals would be expected to decline.

Importantly, the current study models the financial impact of livestock schistosomiasis on a representative herd or flock in the study areas. This study is based on common practices as reported by farmers and reflects a common situation in a regular production year, where there are no major droughts, epidemic outbreaks or similar events. Consequently, the models capture only a narrow set of the infinite possibilities of impact defined by a diverse set of farmers, practices, circumstances, and seasonal and annual fluctuations (caused by weather, celebrations, festive periods, etc.). Further, the input values are based on a wide range of sources and assumptions, as the primary data collected did not cover all aspects sufficiently. For example, limitations were encountered when asking questions about herd size, during which several farmers seemed to give inconsistent answers. This was likely because talking about herd size is taboo based on the belief that talking about it may attract bad luck. This was also found in other studies; for example, Parisse encountered a similar problem of receiving inconsistent or approximate numbers with regard to herd size [55].

The respondents in the current study included transhumance subsistence farmers who rarely kept records. For instance, the mortality rate could not be determined, as the farmers gave no or inconsistent answers to this question. Similarly, the effect on feed use remained inconclusive. The milk yield produced with and without schistosomiasis could not be accurately determined, as respondents typically did not measure the quantity of milk their animals produced or that the household consumed. We also recognised, particularly in the northern Richard Toll regions, that *Fasciola* could be a confounding factor in the diagnosis of the disease, as many of the farmers reported signs that are attributable to liver fluke and other diseases that we could not always identify. To address these limitations in input parameters, other sources were consulted including related studies, scientific literature and expert opinion. Moreover, sensitivity analyses were conducted to assess the influence of uncertain parameters on the financial impact.

Given the limitations of the cross-sectional dataset in this study, we recommend a longitudinal study design with testing of livestock to determine their schistosomiasis status and the recording of the production, treatment and management data. The generation of such baseline data for livestock populations in Senegalese transhumance and subsistence populations can only be achieved with appropriate investment, but funding for NTDs in livestock is scarce [56–58]. There seems to be a general lack of studies of production and economic studies in these settings, a problem most likely exacerbated by a shortage of animal health and One Health economists in the region that could generate knowledge on herd and production data, effects of schistosomiasis in livestock, and health-seeking behaviour. This shortage of capability and capacity will need longer-term investment in education, research and development.

Schistosomiasis is a disease that has a dual burden on human and animal health, and several studies have suggested the role the environment plays in the transmission and hybridisation of the species [16, 59, 60]. A more holistic analysis of the impacts of the disease using One Health economics is recommended in the future to assess the monetary and non-monetary impacts. Practical methods to evaluate the disease costs for zoonotic diseases may include evaluating the net cost of the disease to all sectors, calculating the separable costs for the human health and veterinary sectors, estimating the costs and benefits of an integrated intervention such as treating livestock schistosomiasis, and analysis of the zoonotic disability-adjusted life year (zDALY) [61].

The current study highlights the financial impact livestock schistosomiasis has on traditional subsistence and transhumance farmers keeping cattle, sheep or goats in northern Senegal. The presence of disease and its effects underscore the need to consider livestock schistosomiasis in control programmes. Since the benefits reaped from the treatment of livestock zoonotic infections also spill over into the public health and medical sectors, albeit at a cost to the agricultural sector, multisectoral collaboration will be needed.

## Supplementary Information

**Additional file 1.** Livestock population from all households surveyed.

**Additional file 2.** Focus group questions guide.

**Additional file 3.** Breeds kept by households.

**Additional file 4.** Production types based on predominant breed (local breed).

## Data Availability

All data generated or analysed during this study are included in this published article and its additional files. Other datasets used and/or analysed can be made available by the corresponding author on reasonable request.
