# Supplementary material for: Estimating the financial impact of livestock schistosomiasis on traditional subsistence and transhumance farmers keeping cattle, sheep and goats in northern Senegal
Source: Parasit Vectors. 2022 Mar 22;15:101. doi: 10.1186/s13071-021-05147-w (PMC8938966; doi:10.1186/s13071-021-05147-w)
Supplement: Supplementary file 1 — Additional file 1. Livestock population from all households surveyed. [file 13071_2021_5147_MOESM1_ESM.docx]

**Supplementary Information 1: Livestock Population from all Households Surveyed**

| Location/  Breeds | **Cattle** | | | | **Sheep** | | | | **Goats** | | | |  |
| --- | --- | --- | --- | --- | --- | --- | --- | --- | --- | --- | --- | --- | --- |
|  | Male | | Female | | Male | | Female | | Male | | Female | |  |
|  | Adult | Young | Adult | Young | Adult | Young | Adult | Young | Adult | Young | Adult | Young |  |
| **Barkedji** | | | | | | | | | | | | | |
| Local | 210 | 194 | 847 | 370 | 300 | 779 | 2255 | 421 | 180 | 221 | 842 | 292 |  |
| Cross | 1 | 3 | 11 | 3 | 0 | 0 | 0 | 0 | 4 | 6 | 10 | 5 |  |
| Exotic | 6 | 31 | 13 | 17 | 0 | 0 | 0 | 0 | 0 | 0 | 0 | 0 |  |
| **Lac de Guiers** | | | | | | | | | | | | | |
| Local | 78 | 92 | 539 | 199 | 144 | 67 | 351 | 121 | 36 | 81 | 318 | 161 |  |
| Cross | 10 | 6 | 14 | 9 | 0 | 0 | 0 | 0 | 15 | 19 | 24 | 31 |  |
| Exotic | 0 | 0 | 6 | 5 | 0 | 0 | 0 | 0 | 0 | 0 | 0 | 0 |  |
| **Total** | | | | | | | | | | | | | |
| Local | 288 | 286 | 1386 | 569 | 444 | 846 | 2606 | 542 | 216 | 302 | 1160 | 453 |  |
| Cross | 11 | 9 | 25 | 1 | 0 | 0 | 0 | 0 | 19 | 25 | 34 | 36 |  |
| Exotic | 6 | 31 | 19 | 22 | 0 | 0 | 0 | 0 | 0 | 0 | 0 | 0 |  |
