# Supplementary material for: Estimating the financial impact of livestock schistosomiasis on traditional subsistence and transhumance farmers keeping cattle, sheep and goats in northern Senegal
Source: Parasit Vectors. 2022 Mar 22;15:101. doi: 10.1186/s13071-021-05147-w (PMC8938966; doi:10.1186/s13071-021-05147-w)
Supplement: Supplementary file 2 — Additional file 2. Focus group questions guide. [file 13071_2021_5147_MOESM2_ESM.docx]

**Supplementary Information 2: Focus Group Questions Guide**

**Animal diseases- General**

1. What are the most important signs that your animals are sick?
2. When do you consider animal disease to be a problem for your household? What’s the worst that could happen?
3. What diseases or symptoms in your animals would you treat them for? **Fill in disease sheet**
4. If you have a sick animal, what do you do about it (and then give examples such as: get a diagnosis, treat it, let it get better on its own)?

**Animal diseases- Bilharzia**

1. Do you know of Bilharzia in animals? If so, what are the symptoms?
2. What would be the worst problem you may face if your animal has bilharzia?
3. How long do people usually wait before treating their animals?
4. At what point do people decide to do something for their animal’s Bilharzia? For instance, would they treat them if they were *only* diagnosed with Bilharzia? How much time do people usually wait before treating their sick animals?
5. If one animal tested positive for Bilharzia is it important to treat just the one animal, or all of them? Do some people chose not to treat Bilharzia in animals? Does this matter or is there a point in which they really have to treat their animals?
6. Can people get Bilharzia from their livestock? Can people or their animals get Bilharzia again once they’ve been treated for it?

**Selling and Buying Animals**

1. What is the current market price to sell a young sheep to be slaughtered/killed vs breeding (male and female)? What about for the adult sheep (Male and Female) How about buying? How much does this change if they are sick with Bilharzia? **You must collect information for young and old, male and female, buying and selling, slaughtering vs breeding. Get specific prices for each point.**
2. What is the current market price for a young goat to be slaughtered/killed vs breeding (male and female)? How much does this change if they are sick with Bilharzia? What about for the adult goats (Male and Female)? How about selling? **You must collect information for young and old, male and female, buying and selling, slaughtering vs breeding. Get specific prices for each point.**
3. What is the current market price for a young cow to be slaughtered/killed vs breeding (male and female)? How much does this change if they are sick with Bilharzia? What about for the adult cows (Male and Female- females already lactating vs those yet to breed)? How about selling? **You must collect information for young and old, male and female, buying and selling, slaughtering vs breeding. Get specific prices for each point.**
4. How do these prices change throughout the year? **Please fill out seasonality calendar**
5. When you sell a sick animal, is the price different? How much lower **(try to get an example price difference)?**

**Milk and Meat**

1. What price do you get for milk (per volume or kg)? **Please fill out seasonality calendar**
2. How do these prices change throughout the year? **Please fill out seasonality calendar**
3. How long do you milk each animal for? **Please fill out seasonality calendar**
4. What make you decide to sell an animal?

**Feed**

1. What kind of feed do you use for cows, sheep and goats and how much does each type of feed cost?
2. Does this change if the animal is for breeding, milking, or meat?
3. How much feed do you use for an average animal?
4. How does the feed of your animals change if they have bilharzia? How much does this change cost? Is it different depending on the time of the year an animal gets ill with bilharzia (in what way)?

**Other Production Data**

1. In any given year, of 10 female goats/sheep/cows, how many would die?
2. In any given year, of 10 female goats/sheep/cows, how many would give birth to one live kid? Would any have twins or triplets?
3. In any given year, of 10 female goats/sheep/cows, how many would give birth to a kid born dead? Would any give birth to a live kid that would die soon afterwards?
4. In any given year, of 10 female goats/sheep/cows, how many would abort?
5. Does this change if the animals has bilharzia?

**Medicine**

1. When you have to buy medicine (for your animals or yourself), what kinds of things do you worry about?

What are the most important things to consider when buying Bilharzia medicine for your livestock?
