# Supplementary material for: Estimating the financial impact of livestock schistosomiasis on traditional subsistence and transhumance farmers keeping cattle, sheep and goats in northern Senegal
Source: Parasit Vectors. 2022 Mar 22;15:101. doi: 10.1186/s13071-021-05147-w (PMC8938966; doi:10.1186/s13071-021-05147-w)
Supplement: Supplementary file 3 — Additional file 3. Breeds kept by households. [file 13071_2021_5147_MOESM3_ESM.docx]

**Supplementary Information 3: Breeds kept by Households**

| Cattle | Number (percentage)  n = 80 |
| --- | --- |
| Single breeds  Local  Cross  Exotic  Mixed breeds  Local + Cross  Local + Exotic  Cross + Exotic | 63 (79)  2 (3)  0 (0)  2 (3)  2 (3)  1 (3) |
| Sheep | Number (percentage)  n = 80 |
| Single breeds  Local  Cross  Exotic  Mixed breeds  Local + Cross  Local + Exotic  Cross + Exotic | 59 (74)  0 (0)  0 (0)  0 (0)  0 (0)  0 (0) |
| Goats | Number (percentage)  n = 80 |
| Single breeds  Local  Cross  Exotic  Mixed breeds  Local + Cross  Local + Exotic  Cross + Exotic | 60 (75)  2 (3)  0 (0)  0 (0)  0 (0)  0 (0) |
