# Supplementary material for: Estimating the financial impact of livestock schistosomiasis on traditional subsistence and transhumance farmers keeping cattle, sheep and goats in northern Senegal
Source: Parasit Vectors. 2022 Mar 22;15:101. doi: 10.1186/s13071-021-05147-w (PMC8938966; doi:10.1186/s13071-021-05147-w)
Supplement: Supplementary file 4 — Additional file 4. Production types based on predominant breed (local breed). [file 13071_2021_5147_MOESM4_ESM.docx]

**Supplementary Information 4: Production Types based on Predominant Breed (Local breed)**

| Local breed of Cattle | Number of respondents (percentage)  n = 63 |
| --- | --- |
| Meat, Dairy, Breeding  Dairy, Breeding  Breeding  Meat, Breeding  Meat, Dairy  Meat  N/A | 26 (41)  19 (30)  3 (5)  2 (3)  1 (2)  1 (2)  11 (17) |
| Local breed of Sheep | Number of respondents (percentage)  n = 59 |
| Meat, Dairy, Breeding  Dairy, Breeding  Meat, Breeding  Breeding  Meat  N/A | 20 (34)  13 (22)  7 (12)  5 (8)  1 (2)  13 (22) |
| Local breed of Goats | Number of respondents (percentage)  n = 60 |
| Meat, Dairy, Breeding  Dairy, Breeding  Meat, Breeding  Breeding  Meat, Dairy  N/A | 21 (35)  15 (25)  11 (18)  2 (3)  1 (2)  10 (17) |
